# Effects of Oscillation Amplitude Variations on QCM Response to Microspheres of Different Sizes

**DOI:** 10.3390/s23125682

**Published:** 2023-06-18

**Authors:** Emiliano Zampetti, Maria Aurora Mancuso, Fabrizio Dirri, Ernesto Palomba, Paolo Papa, Alessandro Capocecera, Andrea Bearzotti, Antonella Macagnano, Diego Scaccabarozzi

**Affiliations:** 1 Institute of Atmospheric Pollution Research—National Research Council (IIA—CNR), Research Area of Rome 1, Strada Provinciale 35d, 9-00010 Montelibretti, Italyp.papa@iia.cnr.it (P.P.);; 2 National Institute for Astrophysics INAF-IAPS, Via del Fosso del Cavaliere 100, 00133 Rome, Italy; 3 Mechanical Department, Polytechnic University of Milan, Via La Masa 1, 20156 Milano, Italy

**Keywords:** QCM, sensors, particulate matter, microspheres, microparticles size discrimination

## Abstract

Suspended particulate matter (PMx) is one of the most important environmental pollutants. Miniaturized sensors capable of measuring and analyzing PMx are crucial in environmental research fields. The quartz crystal microbalance (QCM) is one of the most well-known sensors that could be used to monitor PMx. In general, in environmental pollution science, PMx is divided into two main categories correlated to particle diameter (e.g., PM < 2.5 µm and PM < 10 µm). QCM-based systems are capable of measuring this range of particles, but there is an important issue that limits the application. In fact, if particles with different diameters are collected on QCM electrodes, the response will be a result of the total mass of particles; there are no simple methods to discriminate the mass of the two categories without the use of a filter or manipulation during sampling. The QCM response depends on particle dimensions, fundamental resonant frequency, the amplitude of oscillation, and system dissipation properties. In this paper, we study the effects of oscillation amplitude variations and fundamental frequency (10, 5, and 2.5 MHz) values on the response, when particle matter with different sizes (2 µm and 10 µm) is deposited on the electrodes. The results showed that the 10 MHz QCM was not capable of detecting the 10 µm particles, and its response was not influenced by oscillation amplitude. On the other hand, the 2.5 MHz QCM detected the diameters of both particles, but only if a low amplitude value was used.

## 1. Introduction

Air pollution is an increasingly important problem that affects human life, impacting health, sociocultural, and economic aspects [1,2]. There are many sources of pollution, and they are distributed in different ways on our planet. However, air pollutants emitted in one country can be transported into the atmosphere, affecting air quality elsewhere. Suspended particulate matter (PMx), nitrogen dioxide, and tropospheric ozone are considered the three pollutants that most significantly affect human health [3]. For these reasons, PMx measurement is very important, and can be performed using various direct analytical instruments, such as impact samplers and analytical balances [4]. In recent years, systems and sensors for the analysis (or counting) of microparticles or microspheres have been the focus of numerous studies. For the assay, coated microspheres provide a measuring tool in biology and drug research [5]. In buoyancy, hollow microspheres (made of glass and polymer) are used to decrease material density in plastics. For ceramic filter manufacturing, microspheres of polyethylene are used to obtain controlled porosity [6]. In particular, in the field of research for developing PM monitoring devices, monodisperse microspheres are used to calibrate particle sieves, optical particle counters (OPCs), and other kinds of sensors [7]. Generally, OPC performances are comparable when they are used to count particles. On the other hand, the algorithms used to convert the counting into a mass concentration differ from manufacturer to manufacturer. In fact, parameters such as refractive index and particle shape influence their mass estimation. Therefore, the optical measurement has an intrinsic discrepancy when compared to the more accurate direct gravimetric methods [8]. For these reasons, we focused our attention on quartz crystal microbalances (QCMs), which are one of the most commonly used mass sensors for the development of sensory systems designed to measure gases, molecules, particles, and contaminants in a wide range of applications, such as environmental monitoring [9], medical assays, and space applications [10,11,12,13]. QCMs are piezoelectric oscillating-based sensors capable of converting changes in thickness/mass of the deposited material on their electrodes into a frequency shift. QCMs are still being studied as one of the most reliable methods of measurement and analysis for particulate matter in general [14,15,16]. In fact, in PM monitoring, a QCM can measure the mass of the particles collected on its electrodes by providing, for example, the total of PM or fractions such as PM2.5 or PM10 in cascade-type impact meters [17]. Separation or filtering systems [18] and one or more QCM are therefore used to obtain information on the different dimensions of the particulate. Currently, this type of system can be miniaturized, but it poses extreme difficulties given the current state of microfluidics, mechanical, or 3D machining processes [19]. In recent years, QCMs have been at the center of numerous research studies applied to the measurement of the dimensions of microspheres in various fields of application; in fact, many works use them not only as mass sensors, but also as actual transducers for complex analyses of tribology [20,21]. Some scientific works use concepts of energy dissipation of the vibration generated on the surface of the QCM to analyze the type of bond the particle forms with the electrode surface, often functionalized [22]. Other research groups modify the surface of the QCM by building mechanical structures, such as micropillars, that act as mechanical resonators and, when coupled with particles of the correct size, produce changes in the frequency of the QCM distinguishable from the simple signal of an increase in mass or thickness [23]. However, it would be very interesting to be able to use QCMs to measure, at the same time, the mass of particles collected on the surface and perform dimensional analysis. This would prevent PM monitoring systems, based on mass sensors, from always having to use complex or cumbersome dimensional separation systems. In 1971, a study by Olin and Sem [24] was useful in understanding how to write a relationship between the response of a QCM, the resonance frequency, and the amplitude of the vibration when microparticles are deposited on its surface. In this paper, we present an experiment aimed at studying the possible effects that occur on the response of a QCM to different measurements of microspheres when the amplitude of the vibration varies. To simplify the tests, we used borosilicate microspheres, which are usually used in aerosol generators, for atmospheric PM simulation. In detail, we conducted tests on various QCMs, which had three distinct fundamental resonant frequencies (10, 5, 2.5 MHz), measuring the response obtained for two samples of microspheres, 2 and 10 µm in diameter, and varying the amplitude of the vibration.

We have obtained encouraging results by developing a circuit capable of controlling the amplitude of the oscillation, acting on the QCM driving current. In particular, with a QCM of 2.5 MHz, we can detect only the mass of the 2 µm microspheres, even when 10 µm microspheres are present. This result confirms Olin and Sem’s study, and could allow the development of a QCM-based system for continuous mass and size analysis of collected particulate matter.

## 2. Methods and Materials

### 2.1. Frequency and Driving Force Effects

According to Sauerbrey’s experimental assumption, the resonant frequency decreases linearly with the addition of foreign mass on the crystal surface:

(1) $$\Delta f=-\frac{2{fo}^{2}}{A\sqrt{v_{q}\rho_{q}}}\Delta m=-\frac{C_{f}}{A}\Delta m$$

In this equation (Equation (1)), Δ*f* refers to the frequency shift due to the changing mass Δ*m*. *C_f_* represents a constant, where *f*_0_ is the fundamental resonating frequency, *v_q_* is the velocity of propagation of transverse wave in the plane of the quartz, *ρ_q_* is the density of the quartz, and *A* is the effective area of the electrode [25].

Although the Sauerbrey assumption is applicable for particles and thin films, some differences in the QCM response were observed due to particles that may have relatively more movement between adjacent particulate layers, causing more energy dissipation [26]. Generally, a particle on a QCM must adhere to the vibrating surface with enough force to counteract the inertia forces to be detected [24,27,28,29].

The inertia force *F_I_* acting on a spherical particle of diameter *D_p_* and density *ρ_p_* is defined as:

(2) $$F_{I}=\rho_{P}\left(\frac{\pi }{6}\right)\beta I({2\pi f_{0})}^{2}{D_{p}}^{3}$$

where *βI* is the crystal vibration amplitude for a fixed driving current *I*, and *f*_0_ is the crystal fundamental resonating frequency (*β* is a constant for a given crystal). The particle adhesion force (*F_A_*) on the surface can be written as:

(3) $$F_{A}=\mu \lambda D_{p}$$

where *µ* is the friction coefficient and λ is a constant depending on the interaction forces between the surface and the particle. When the forces are balanced (*F_A_ = F_I_*), combining Equations (2) and (3), we obtain a critical diameter *D_crit_* (for a fixed frequency and a set driving current *I*):

(4) $$D_{crit}=\sqrt{\frac{3\mu \lambda }{{2\pi }^{3}\rho_{P}\beta }}\left(\frac{1}{I^{1/2}f_{0}}\right)$$

Equation (4) shows that when *F_I_ ≤ F_A_,* a particle adheres to the QCM surface, resulting in $D_{p}$ ≤ $D_{crit}$, and it can be detected. On the other hand, it cannot be detected when *F_I_ > F_A_* because a particle tends to dislodge from the surface ($D_{p}$ > $D_{crit})$. For these reasons, varying the current and frequency makes it possible to change the QCM response to a fixed particle diameter. In order to detect bigger particles, the product *I∙f*_0_ should be minimized. However, there are physical limits for the driving current and resonating frequency. In particular, the current cannot be less than *I_Limit_* (the value required to sustain a stable oscillation), and at the same time, a decreasing resonant frequency produces a QCM response (or sensitivity) reduction.

### 2.2. Reagents and Methods

The chemicals used to prepare the samples, which included three dispersions and one solution, were polyvinylpyrrolidone (MW 1300 kDa, CAS number: 9000-39-8), absolute ethanol (CAS number: 64-17-5) purchased from Merck (Darmstadt, Germany), and borosilicate glass microspheres with mean diameters of 2 µm and 10 µm (traceable to the National Institute of Standards and Technology) purchased from Thermo Scientific (Waltham, MA, USA) (Cat.no:9002, Cat.no:9010).

Three different dispersions were prepared using absolute ethanol (EtOH) as liquid phase [30]. MS_10_ contained 8.5 mg of 10 µm microspheres (d = 2.55 g/cm^3^) in 10 mL of EtOH (0.89 µg/µL), MS_2_ contained 3 mg of 2 µm (d = 2.50 g/cm^3^) microspheres in 10 mL of EtOH (0.30 µg/µL), and MS_MIX_ contained 2.1 mg of 10 µm microspheres and 1.4 mg of 2 µm microspheres in 10 mL of EtOH, resulting in concentrations of 0.42 µg/µL and 0.28 µg/µL, respectively. These dispersions were used to test the response of QCMs using a drop-casting technique, depositing a volume of 1 µL on their surface. Before each deposition, the dispersions were sonicated in an ultrasound bath for 3 min to avoid precipitation. In addition, to compare the influence of oscillation amplitude on the QCM response in the presence of a polymeric homogenous film versus microsphere dispersions, a PVP solution (PVP_sol_) was prepared using EtOH as solvent (0.18 µg/µL).

**Figure 1:**
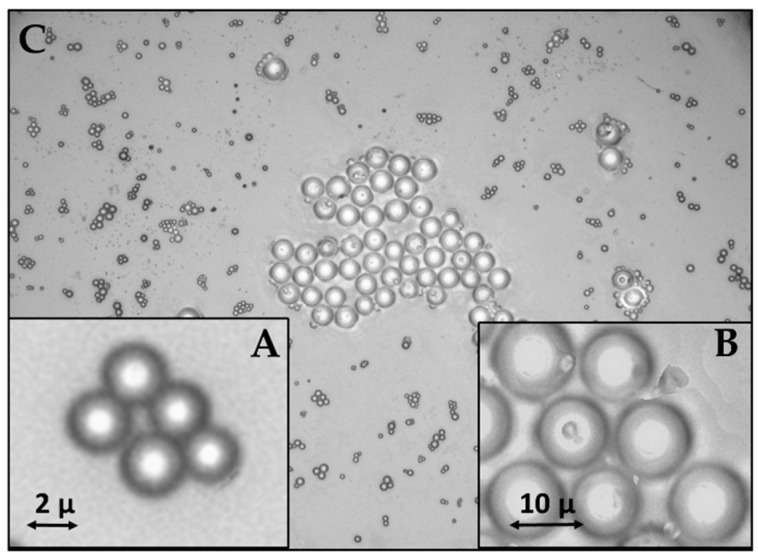
Microscope images of borosilicate glass microspheres in MS_2_ (**A**), MS_10_ (**B**), and MS_MIX_ (**C**) dispersions deposited over a quartz crystal slice.

Using an optical microscope (Leica DM2700 M) with an objective of 100×/0.85 magnification and a field of view of 22 mm, it was possible to observe three depositions of borosilicate microspheres in the different dispersions (MS_10_, MS_2_, MS_MIX_) on a quartz crystal slice, as shown in Figure 1.

Three types of QCMs (10 MHz, 5 MHz, and 2.5 MHz of resonance frequency) were used, each with a different gold-coated electrode size (d = 5.9 mm, d = 6.3 mm, d = 5.0 mm, respectively), to evaluate the changes in responses due to variations in the fundamental frequency (as shown in Equation (4)). Moreover, we used a specific QCM (with a fixed fundamental frequency) to measure the response of each sample (MS_10_, MS_2_, MS_MIX_, PVP_sol_), using three levels of driving current, I_LOW_, I_MED_, and I_H_ (to change the oscillation amplitude).

### 2.3. Measurement Setup

**Figure 2:**
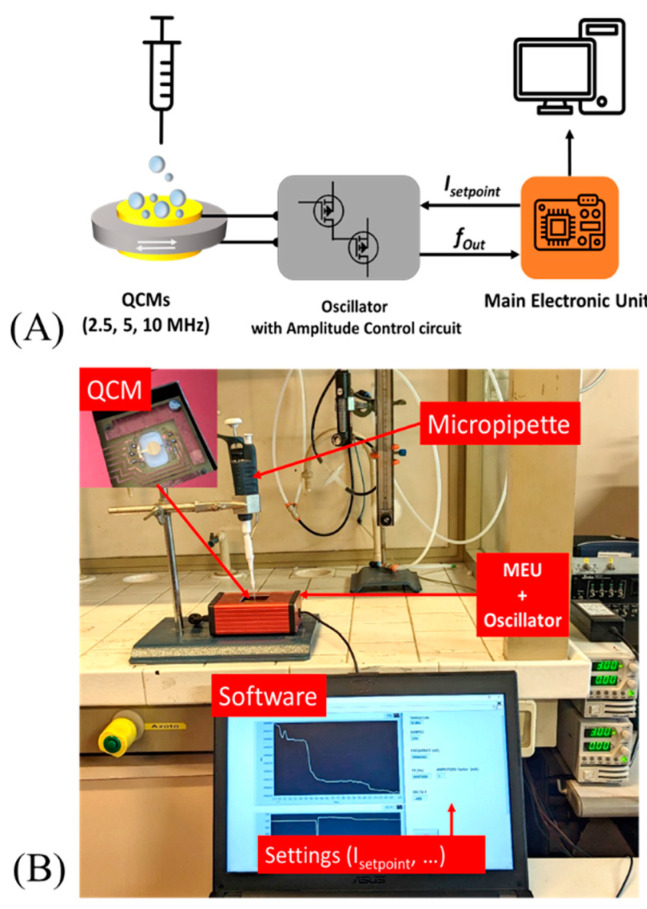
Schematic view of measurement setup used during the experiments (**A**). Photograph of the measurement setup used during the experiments (**B**).

A block diagram of the measurement setup used during all of the experiments is represented in Figure 2A. A micropipette (PIPETMAN P2, variable volume 0.2–2.0 µL, Serial N. KB29151) was used to deposit the sample of microsphere dispersion onto the QCM electrode by drop-casting. To simplify and reduce some errors, due to mechanical aspects, the micropipette was mechanically fixed on a robust stand during all depositions.

As previously described, we used three different QCMs to carry out all of the presented tests. To operate, a QCM needs to be connected to an oscillator circuit that sustains the oscillation conditions [31].

In Figure 2B, we have a photograph of the measurement setup where the highlighted QCM sensor (in particular, a 10 MHz crystal is in the photo) is connected to an oscillator circuit and to the main electronic unit, both enclosed in a single box (red one). A developed software controlled the measurement acquisitions and set all experimental parameters.

In this work, we used a suitable QCM oscillator circuit capable of sustaining the oscillation, and to set the crystal current (oscillator with amplitude control circuit, AC-OSC). Although in this paper we will not go into detail on how it works from an electronics point of view (as the circuit is under patent), the AC-OSC consists of two main parts, each working separately to sustain the oscillation (part a) and to control the amplitude of vibration by changing the oscillation current (part b). As already mentioned above, there is a lower limit current (*I_Limit_*) required for a stable oscillation, which depends on different QCM parameters (e.g., drive power level limit). In all of the experiments, we used three different setpoints of current for each QCM: low amplitude (Low), medium amplitude (Med), and high amplitude (High) correlated to the crystal current. In particular, about 0.9 mA, 1.8 mA, and 3.5 mA were used for the low, medium, and high amplitude, respectively (the crystal current is expressed by its root mean square value—RMS).

The main electronic unit (MEU) consisted of a microcontroller that managed all of the measurement phases, setting the driving current, acquiring the signal (*f_out_*) with a LOD of ±3 Hz, and sending the measurement data to the PC unit for plotting and storage. Environmental information, such as temperature and relative humidity, were measured by a DHT22 sensor connected to µC serial bus.

## 3. Results and Discussions

**Figure 3:**
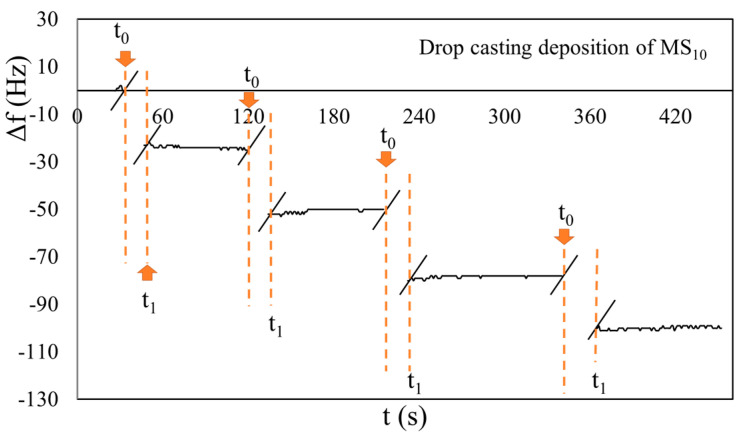
Chronogram of four MS_10_ depositions of the same volume (1 μL) on a 2.5 MHz QCM. Oblique lines “//” indicate the time intervals where the signal was not stable due to the deposition effects. The t_0_ and t_1_ are the instant of the beginning of deposition and when the signal returned stable, respectively.

**Figure 4:**
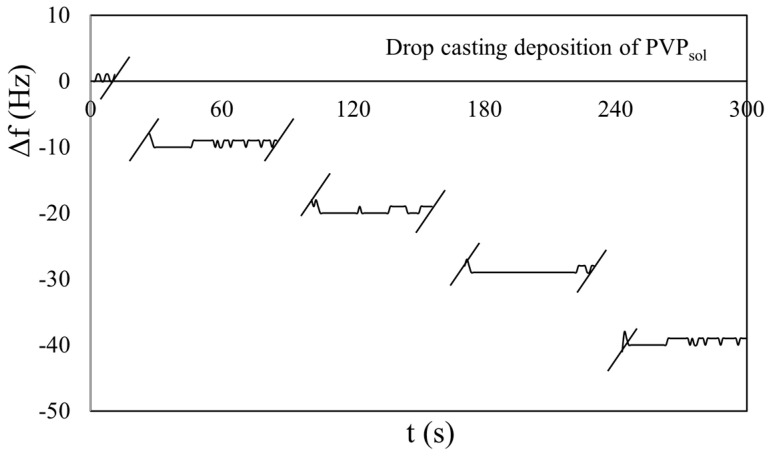
Chronogram of PVP_sol_ by injecting the same volume (1 μL) on a 2.5 MHz QCM.

In the beginning, different depositions of the same volumes of PVP_sol_ and MS_10_ on QCMs were performed to confirm the linear behavior of the Sauerbrey equation (Equation (1)). Examples of the obtained results, in the case of the 2.5 MHz crystal, are shown in Figure 3 and Figure 4. To simplify the arrangement of the plot in all chronograms shown in this paper, we have not reported the dynamic behavior of the response |Δ*f*| during the drop-casting deposition. In fact, at the beginning of the deposition (t_0_), the response fluctuated between 1 and 2 kHz, and returned stable (t_1_) after tens of seconds. These phenomena could have been produced by the injection pressure and liquid phase of the samples. To highlight this time interval, we have introduced two oblique lines “//” in the plot, and only in the first have we indicated both t_0_ and t_1_ instants for each deposition.

Essentially, no appreciable discrepancies were observed in the frequency response |Δ*f*| between consecutive depositions. The QCMs exhibited linear and repeatable responses for each deposition. To highlight the results, only a single deposition example will be shown for each value of the set oscillation amplitude (Low, Med, High) in all of the following chronograms.

### 3.1. Homogeneous Film of PVP Deposition

**Figure 5:**
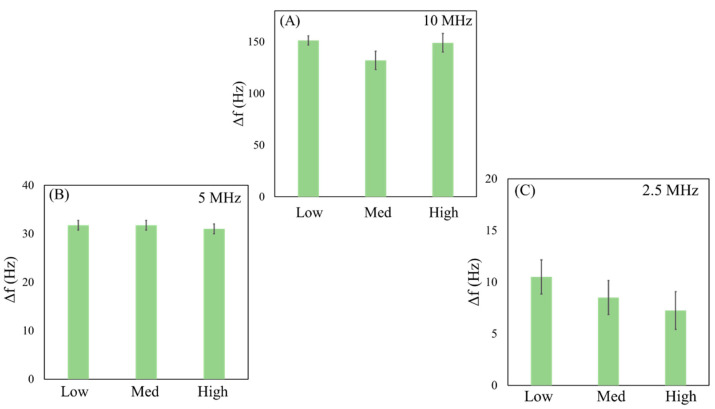
Histograms of PVP_sol_ with different amplitude levels (Low, Medium, High) on 10 MHz (**A**), 5 MHz (**B**), and 2.5 MHz (**C**) QCMs.

Ten tests of a single deposition of PVP_sol_ (1 µL) were performed to investigate the effects of amplitude variations on the response of the QCMs. The results of all tests are summarized in the histograms in Figure 5. The same test procedure was executed for all three dispersions (MS_2_, MS_10_, and MS_MIX_).

In Figure S1 of the Supplementary Materials, we report an example of the results obtained after PVP_sol_ deposition on 10 MHz, 5 MHz, and 2.5 MHz crystals, carried out at low, medium, and high amplitude values. When the driving force varies (due to changes in amplitude), frequency shifts are significantly similar for each QCM. No differences in calculated mass (Equation (1)) were observed, despite varying resonance frequency and amplitude levels. The theoretical mass value (180 ng) is very close to the experimental masses obtained, according to PVP*_sol_* concentration. The results are summarized in Table S1 of the Supplementary Materials. These data are in agreement with the thin film behavior reported in the literature [32,33,34,35]. Thin films are deposited as homogeneous layers onto the QCM electrode, and the frequency changes are related to an increase in mass, as predicted by the Sauerbrey equation (Equation (1)). In this experiment, the deposition on the electrode of the same volume of PVP leads to the formation of a homogeneous layer. The response does not vary appreciably by changing the *f*_0_ and the amplitude. Table 1 shows the average values of frequency calculated from all of the tests performed.

### 3.2. MS_2_ (Microspheres of 2 µm) and MS_10_ (Microspheres of 10 µm)

Examples of chronograms for MS_2_ dispersions are shown in Figure S2 in the Supplementary Materials for QCMs operating at 10 MHz (A), 5 MHz (B), and 2.5 MHz (C) frequencies, with low, medium, and high amplitudes. Even in this case, a behavior similar to PVP_sol_ can be observed at the amplitude variation. Frequency changes are related to very similar experimental masses when different QCMs are used, according to MS_2_ concentration. These results are reported in Table S2. No notable changes in the frequency variation were observed by modulating the amplitude for each sensor. According to Equation (4), the particle diameter could be below the critical dimension for these three different levels (*D_p_ = 2* μm < *D_crit_)*. We have evaluated the deposited mass for each QCM by Equation (1), taking into account the fundamental resonating frequency (10 MHz, 5 MHz, 2.5 MHz) [36] and the frequency shifts (|Δ*f*|) reported in Table S2. The calculated mass values were in the range of 256.6 ng to 329.5 ng, with tens of nanograms of deviation from a theoretical mass of 300 ng. Examples of chronograms for MS_10_ dispersions are shown in Figure S3 in the Supplementary Materials for QCMs operating at 10 MHz (A), 5 MHz (B), and 2.5 MHz (C) frequencies with low, medium, and high amplitudes. The frequency shifts (|Δf|) are reported in Table S3.

**Figure 6:**
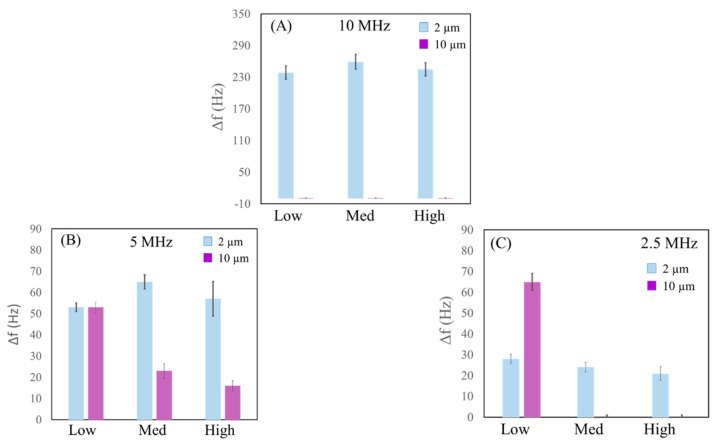
Histograms of MS_2_ and MS_10_ in EtOH with different levels of amplitude (low, medium, high) on 10 MHz (**A**), 5 MHz (**B**), and 2.5 MHz (**C**) QCMs.

Ten tests were performed both for MS_2_ and MS_10_, aiming to compare the results summarized in Figure 6. The histograms were constructed by considering the comparison between the depositions of MS_2_ and MS_10_ on each microbalance, achieved by modifying the amplitude.

In Figure 6A, the statistical dispersion of MS_2_ (blue) confirms that the average frequency variations are related to the concentration of the dispersion where *D_p_ = 2 μm* < *D_crit_*, as expected. No response to any amplitudes is observed on the 10 MHz QCM (purple). As explained in Section 2.1, and as known from the literature [37,38,39], for lower coupling and larger dimensions, the particles are not able to follow the movement with the vibrations of the QCM. On the contrary, for strong coupling and smaller mass, the particles move with the vibrational surface of the QCM. Since the gold surface of the QCM and the borosilicate microspheres do not establish chemical bonds on the contact surface, the adhesion force depends mostly on the particle diameter and friction coefficient (Equation (2)). Experiments for this resonance frequency and the three modulated amplitude oscillations show a particular condition where particle diameter is larger than the critical dimension (*D_p_* = 10 μm > *D_crit_*).

In Figure 6B, the average frequency variations ($\overset{®}{\Delta f}$) are plotted as a function of the amplitudes on the 5 MHz QCM for ten tests carried out. Blue histograms represent the data found on MS_2_ tests, where these average frequencies are once again in agreement with the concentration of the dispersion. There is a possibility that this resonance frequency is critical for MS_10_, where a strong dependence on the levels of amplitude has been observed (*D_p_* ~ 10 μm). In the same way, the purple histograms show a decrease in average frequency variations ($\overset{®}{\Delta f}$ = 16 Hz) when the high level is used (fewer and smaller particles are detected), while the histograms show an increase ($\overset{®}{\Delta f}$ = 53 Hz) when a lower level of amplitude is applied (more and bigger particles are measured), according to Equation (4).

Figure 6C shows the results of $\overset{®}{\Delta f}$ obtained at different levels of oscillation amplitude on the 2.5 MHz QCM. Blue histograms (MS_2_) show no significant differences in the response when modulating the amplitude. All of the tests with MS_10_ showed that the response depends on the amplitude value. In fact, for high and medium levels, no response was observed because *D_p_ > D_crit_*, while for low levels *D_p_ < D_crit_*, a response was obtained. The average frequency variation obtained (65 Hz) was identifiable to the deposited mass (calculated mass was 908.7 ± 74.6 ng). The average frequency variations obtained from the histograms are summarized in Table 2.

### 3.3. MS_MIX_ (Microsphere Mix)

**Figure 7:**
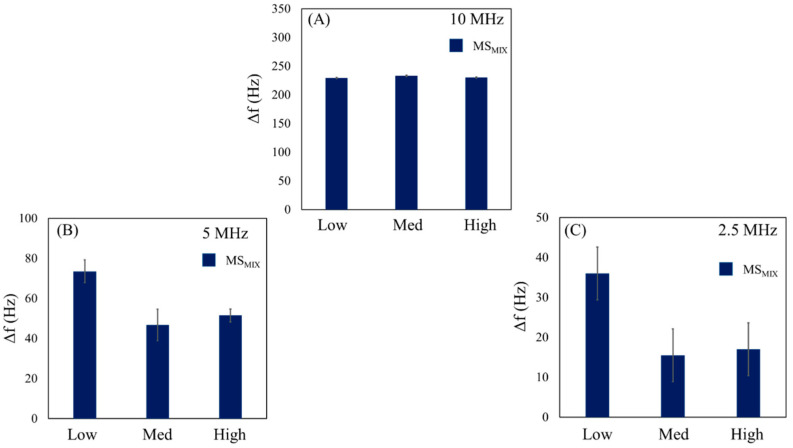
Histograms for MS_MIX_ (2 and 10 µm) in EtOH on 10 MHz (**A**), 5 MHz (**B**), and 2.5 MHz (**C**) QCMs with different oscillation amplitudes.

Examples of chronograms for MS_MIX_ dispersions are shown in Figure S4 in the Supplementary Materials for QCMs operating at 10 MHz (A), 5 MHz (B), and 2.5 MHz (C) frequencies, with low, medium, and high amplitudes. The measured frequency shifts are reported in Table S4. In order to compare the results obtained for MS_10_ and MS_2_, ten tests were performed for MS_MIX_. In Figure 7, we report the histograms of results on each microbalance, achieved by modifying the amplitude.

Figure 7A shows statistical data of tests on a 10 MHz QCM. Considering the responses previously obtained for MS_2_ and MS_10_ dispersions, frequency variations should be assigned to the 2 μm microspheres because no response was observed for MS_10_ (*D_p_* = 10 μm > *D_crit_*). This outcome is consistent with the previous discussion (Section 3.2); the average frequency variations (Table 3) are related to the concentration of these particles in MS_MIX_. In the same way, Figure 7B reports the results of MS_MIX_ on a 5 MHz QCM, where a critical dimensional condition for MS_10_ is observed (*D_crit_* around 10 μm), and a strong dependence on the level of oscillation amplitude is shown. For this reason, when high and medium levels are used, the value of *D_crit_* decreases, and the average frequency variations are related to 2 μm microspheres and smaller particles in the dimensional distribution of 10 μm in MS_MIX_. For low levels, the value of *D_crit_* increases, and 2 μm microspheres with larger particles of 10 μm in MS_MIX_ are detected.

Finally, Figure 7C shows histograms of the tests performed on the 2.5 MHz QCM. The average frequency variation observed should be attributed to the 2 μm microspheres in MS_MIX_, because no response was observed for 10 μm particles (*D_p_* = 10 μm > *D_crit_*) at high and medium levels. However, for low amplitude, the condition where *D_p_* = 10 μm < *D_crit_* was observed, and 2 μm and 10 μm microspheres in MS_MIX_ were detected together. The average frequency variations obtained from the histograms are summarized in Table 3.

## 4. Conclusions

In this paper, we study the effects of oscillation amplitude variations and fundamental frequency value on the QCM response, when particle matter of different sizes is deposited on the electrodes. Four different samples were prepared (PVP_sol_, MS_2_, MS_10_, and MS_MIX_). From the literature, it is known that the detection of microspheres is influenced by resonant frequency (*f*_0_) and crystal vibration amplitude (*βI*). When *D_p_ < D_crit_*, a particle adheres to the QCM surface and can be detected. On the contrary, when *D_p_ > D_crit_*, a particle tends to slip from the surface. For the solution of PVP, no appreciable changes in responses with varying QCMs and amplitude levels were observed, according to homogenous film behavior. Similar performances to PVP_sol_ were observed for MS_2_ dispersion because the particle diameter is reasonably under the critical dimension (*D_p_ = 2* μm < *D_crit_)*. For MS_10_, no response to any oscillation amplitude level was observed on the 10 MHz QCM. When the particle diameter overcomes the critical diameter (*D_p_* = 10 μm > *D_crit_*), the oscillation returns to the fundamental frequency after tens of seconds. A critical resonance frequency was assumed for the 5 MHz QCM, where *D_crit_* was around 10 μm. The response was worsened when a high level of amplitude was applied, and improved when a low level was used. The response of the 2.5 MHz QCM to MS_10_ depended strongly on the applied amplitude levels: high and medium intensities showed *D_p_ > D_crit_*, while for the low level *D_p_ < D_crit_*, and 10 μm microspheres were detected. In the case of MS_MIX_, the responses on the 10 MHz QCM should be assigned to the 2 μm microspheres (*D_p_ = 10 μm > D_crit_*). More elaborate is the case on the 5 MHz QCM, where D_crit_ was around 10 μm and strong frequency variations were observed when different oscillation amplitude levels were applied. On the 2.5 MHz QCM for high and medium levels, the responses should be assigned to the 2 μm microspheres because no response was observed for 10 μm particles. For low levels, 2 μm and 10 μm microspheres were measured together due to the condition where *D_p_* = 10 μm < *D_crit_* was observed. To validate the study carried out in this paper, it would be suitable to study intermediate conditions, considering resonance frequencies from 5 MHz to 2.5 MHz, where microspheres have a dimension between 2 µm and 10 µm for better estimation of the critical diameter value.

This experiment’s results have shown that by changing the oscillation amplitude and fundamental resonant frequency, it is possible to use a single QCM (e.g., 10 MHz) to measure only 2 µm particles when both 2 and 10 µm are collected on the QCM electrode. On the other hand, with a 2.5 MHz QCM, it is possible to measure both particles or only 2 μm, if the amplitude oscillation is changed. These results could be used to develop a QCM array-based monitoring system to carry out particle dimensional analysis, using amplitude modulation.

## Figures and Tables

**Table 1:** The table presents average values of frequency after ten tests for PVP_sol_ on different QCMs (10, 5, 2.5 MHz) and amplitudes (high, medium, low).

QCM (MHz)	Amplitude	$\overset{®}{\Delta f}$ (Hz)	Δf_e_ (Hz)
	High	149.0	± 9.3
10	Medium	135.7	± 7.6
	Low	151.2	± 4.8
	High	31.7	± 4.2
5	Medium	31.7	± 7.6
	Low	31.7	± 4.8
	High	7.2	± 0.8
2.5	Medium	8.5	± 0.6
	Low	10.5	± 0.6

Δf_e_ is the standard deviation of the arithmetic mean of frequency.

**Table 2:** The table presents average values of frequency after ten tests for MS_10_ and MS_2_ on different QCMs (10, 5, 2.5 MHz) and oscillation amplitudes (high, medium, low).

	**QCM (MHz)**	**Driving Force**	$\overset{®}{\Delta f}$ **(Hz)**	**Δf_e_ (Hz)**
2 µm		High	245.0	±12.5
	10	Medium	259.2	±13.8
		Low	238.7	±12.4
		High	52.7	±1.1
	5	Medium	62.2	±2.3
		Low	52.7	±1.1
		High	21.0	±2.3
	2.5	Medium	24.0	±1.2
		Low	27.7	±1.1
10 µm		High	-	-
	10	Medium	-	-
		Low	-	-
		High	16.0	±1.5
	5	Medium	23.2	±2.4
		Low	53.2	±1.4
		High	-	-
	2.5	Medium	-	-
		Low	65.0	±4.1

**Table 3:** The table presents average values of frequency after ten tests for MS_MIX_ on different QCMs (10, 5, 2.5 MHz) and oscillation amplitudes (high, medium, low).

QCM (MHz)	Driving Force	$\overset{®}{\Delta f}$ **(Hz)**	Δf_e_ (Hz)
	High	230.0	±6.0
10	Medium	233.0	±3.9
	Low	229.2	±8.5
	High	51.5	±3.2
5	Medium	46.7	±7.8
	Low	73.5	±5.7
	High	17.0	±1.9
2.5	Medium	15.5	±1.0
	Low	31.0	±1.2

## Supplementary Materials

The following supporting information can be downloaded at: https://www.mdpi.com/article/10.3390/s23125682/s1, Figure S1: Chronograms for PVP solution on 10 MHz (A), 5 MHz (B), and 2.5 MHz (C) QCMs using different amplitude (low, medium, and high). Figure S2: (MS_2_) 2 μm microsphere dispersion chronograms on 10 MHz (A), 5 MHz (B), and 2.5 MHz (C) QCMs with different oscillation amplitudes. Figure S3: (MS_10_) 10 μm microsphere dispersion chronograms on 10 MHz (A), 5 MHz (B), and 2.5 MHz (C) QCMs with different oscillation amplitudes. Figure S4: Chronograms of MS_MIX_ (2 and 10 µm) in EtOH on 10 MHz (A), 5 MHz (B), and 2.5 MHz (C) QCMs with different oscillation amplitudes. Table S1: The table presents the results obtained for PVP_sol_ changing amplitude (high, medium, low) on different QCMs (10, 5, 2.5 MHz). Table S2: Results obtained for MS_2_ modulating driving force (high, medium, low) on different QCMs (10, 5, 2.5 MHz). Table S3: Results obtained for MS_10_ modulating driving force (high, medium, low) on different QCMs (10, 5, 2.5 MHz). Table S4: The table presents frequency variations for MS_MIX_ on different QCMs (10, 5, 2.5 MHz) and oscillation amplitudes (high, medium, low).
