# Supplementary material for: Effects of Oscillation Amplitude Variations on QCM Response to Microspheres of Different Sizes
Source: Sensors (Basel). 2023 Jun 18;23(12):5682. doi: 10.3390/s23125682 (PMC10302074; doi:10.3390/s23125682)
Supplement: Supplementary file 1 [file sensors-23-05682-s001.zip › sensors-2404004-supplementary.pdf]

sensors

Supplementary Materials

Effects of Oscillation Amplitude Variations on QCM Response to Microspheres of Different Sizes

Emiliano Zampetti [1,*], Maria Aurora Mancuso [1], Fabrizio Dirri [2], Ernesto Palomba [2], Paolo Papa [1], Alessandro Capocecera [1], Andrea Bearzotti [1], Antonella Macagnano [1] and Diego Scaccabarozzi [3]

[1] Institute of Atmospheric Pollution Research–National Research Council (IIA-CNR), Research Area of Rome 1, Strada Provinciale 35d, 9-00010 Montelibretti, Italy; aurora.mancuso@iia.cnr.it (M.A.M.); p.papa@iia.cnr.it (P.P.); a.capocecera@iia.cnr.it (A.C.); a.bearzotti@iia.cnr.it (A.B.); antonella.macagnano@cnr.it (A.M.)
[2] National Institute for Astrophysics INAF-IAPS, Via del Fosso del Cavaliere 100, 00133 Rome, Italy; fabrizio.dirri@inaf.it (F.D.); ernesto.palomba@inaf.it (E.P.)
[3] Mechanical Department, Polytechnic University of Milan, Via La Masa 1, 20156 Milano, Italy; diego.scaccabarozzi@polimi.it
* Correspondence: e.zampetti@iia.cnr.it

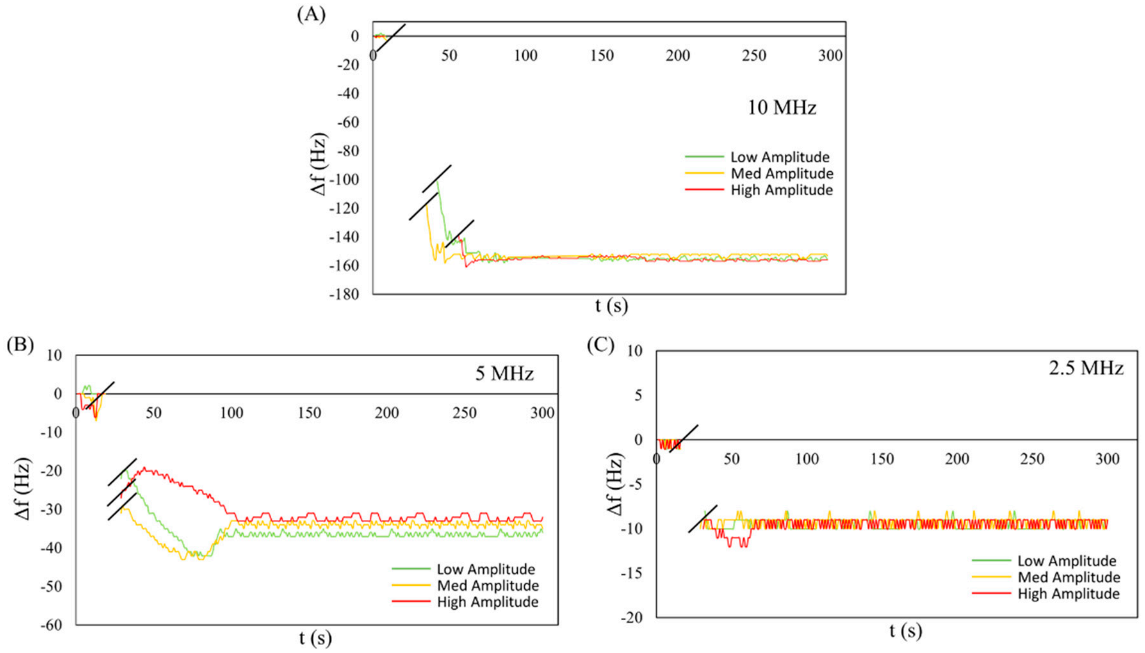

Figure S1. Chronograms for PVP solution on 10 MHz (A), 5 MHz (B), and 2.5 MHz (C) QCMs using different amplitude (low, medium, and high).

Table S1. The table resumes the results obtained for PVP_{sol} changing amplitude (high, medium, low) on different QCMs (10, 5, 2.5 MHz).

QCM (MHz)	Amplitude	ǀΔfǀ (Hz)	Δm (ng)	Δm_e (ng)
10	High	157	196.3	± 9.3
	Medium	149	191.3	
	Low	156	195.1	
5	High	37	164.4	± 20.6
	Medium	34	169.3	
	Low	33	184.3	
2.5	High	10	201.3	± 66.0
	Medium	9	181.2	
	Low	9	181.2	

Δm_e is the experimental mass error.

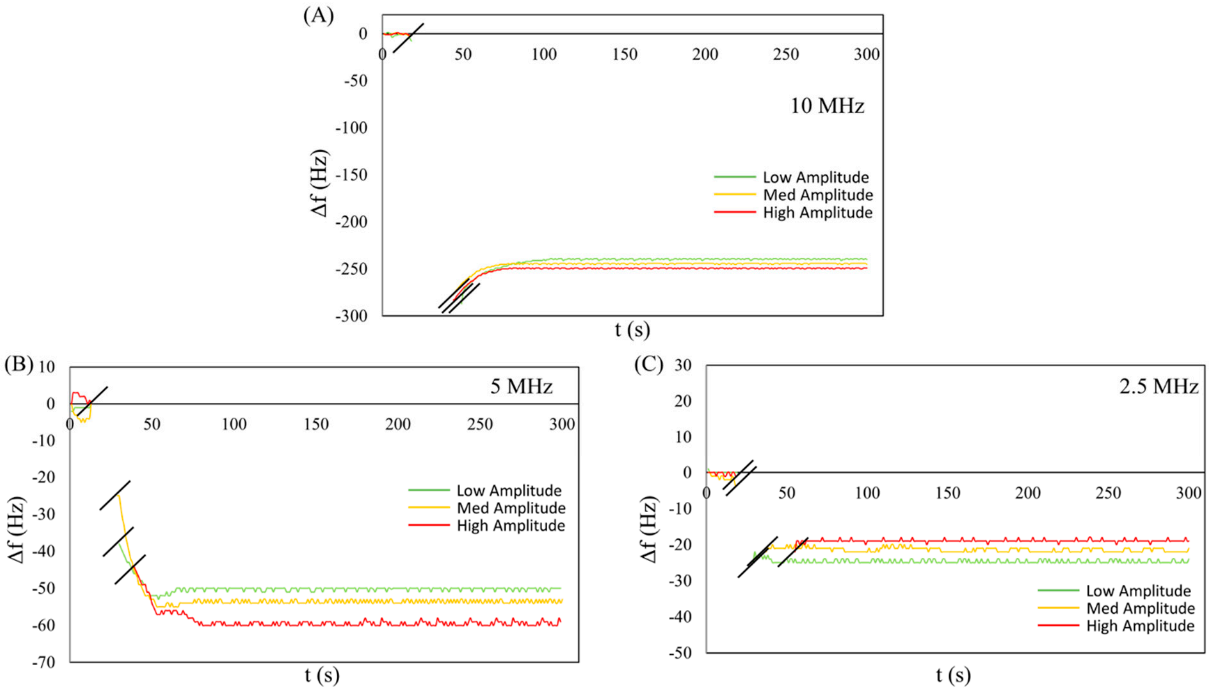

Figure S2. (MS2) 2μm microspheres dispersions chronograms on 10 MHz (A), 5 MHz (B), 2.5 MHz (C) QCMs with different oscillation amplitudes.

Table S2. Results obtained for MS_2 modulating driving force (high, medium, low) on different QCMs (10, 5, 2.5 MHz).

QCM (MHz)	Amplitude	ǀΔfǀ (Hz)
10	High	249
	Medium	245
	Low	240
5	High	58
	Medium	54
	Low	50
2.5	High	19
	Medium	21
	Low	25

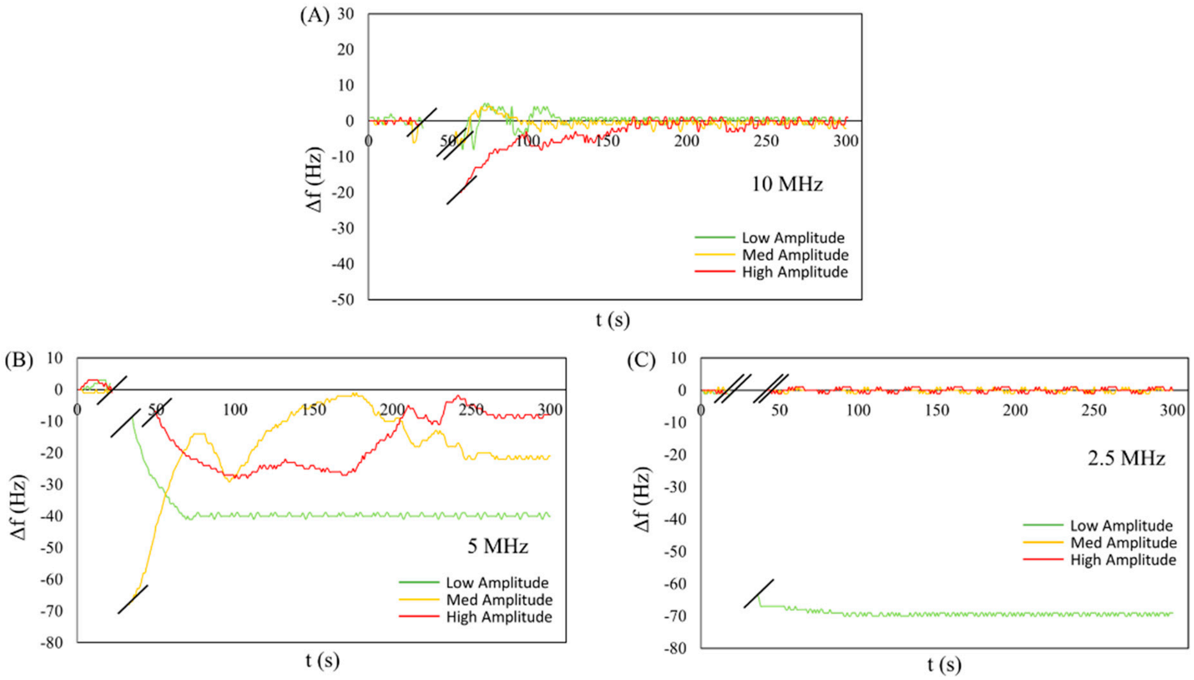

Figure S3. (MS10) 10 μm microspheres dispersions chronograms on 10 MHz (A), 5 MHz (B), 2.5 MHz (C) QCMs with different oscillation amplitudes.

Table S3. Results obtained for MS_{10} modulating driving force (high, medium, low) on different QCMs (10, 5, 2.5 MHz).

QCM (MHz)	Driving force	∣Δf∣ (Hz)
	High	-
10	Medium	-
	Low	-
	High	9
5	Medium	23
	Low	40
	High	-
2.5	Medium	-
	Low	69

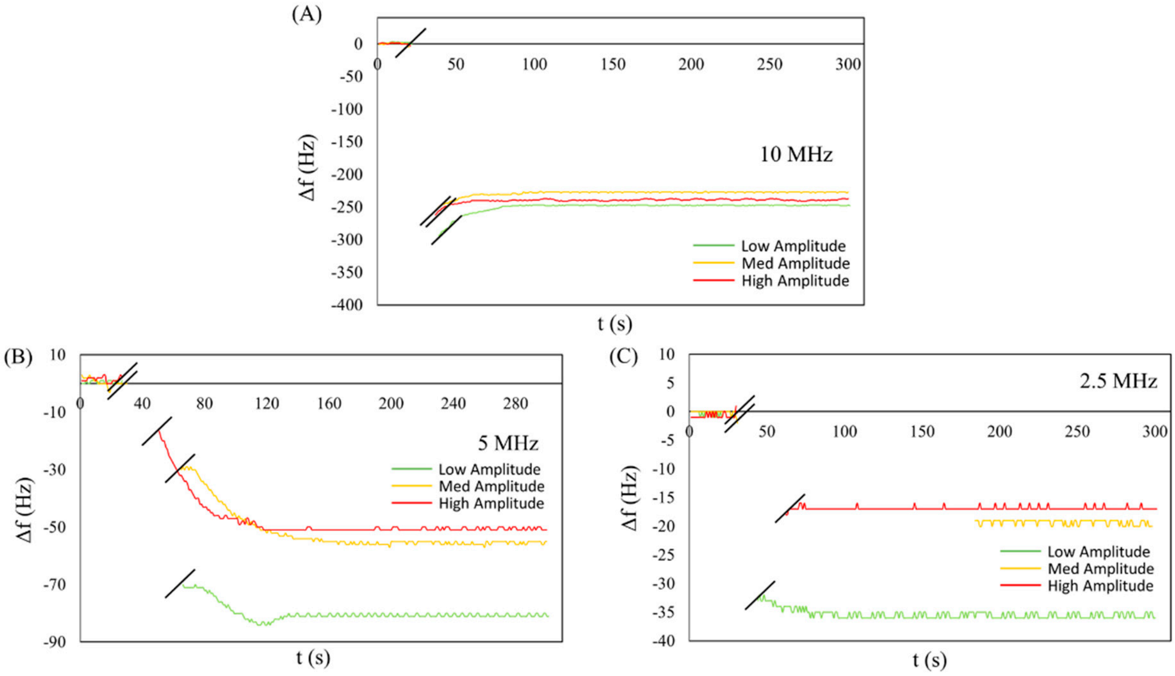

Figure S4. Chronograms of MSMIX (2 e 10 µm) in EtOH on 10 MHz (A), 5 MHz (B), 2.5 MHz (C) QCMs with different oscillation amplitudes.

Table S4. The table resumes frequency variations for MS_{MIX} on different QCMs (10, 5, 2.5 MHz) and oscillation amplitudes (high, medium, low).

QCM (MHz)	Driving force	|Δf| (Hz)
	High	239
10	Medium	227
	Low	247
	High	36
5	Medium	56
	Low	82
	High	16
2.5	Medium	18
	Low	37
